# Exploring mediators of accelerometer assessed physical activity in young adolescents in the HEalth In Adolescents study – a group randomized controlled trial

**DOI:** 10.1186/1471-2458-12-814

**Published:** 2012-09-21

**Authors:** Ingunn H Bergh, Maartje M van Stralen, May Grydeland, Mona Bjelland, Nanna Lien, Lene F Andersen, Sigmund A Anderssen, Yngvar Ommundsen

**Affiliations:** 1 Department of Coaching and Psychology, Norwegian School of Sport Sciences, NO-0886, Oslo, Norway; 2 EMGO Institute for Health and Care Research and Department of Public and Occupational Health, VU University Medical Center, 1081 BT, Amsterdam, the Netherlands; 3 Department of Sport Medicine, Norwegian School of Sport Sciences, NO-0886, Oslo, Norway; 4 Department of Nutrition, Faculty of Medicine, University of Oslo, NO-0316, Oslo, Norway

**Keywords:** Mediation, Adolescents, Accelerometer, Physical activity, Intervention

## Abstract

**Background:**

There is a shortage of information about the factors that mediate physical activity intervention effects which involve youth. The purpose of this study was to examine whether personal, social and physical-environmental factors mediated the intervention effect on physical activity and whether gender and weight status moderated mediated effects in the Health In Adolescents Study – a school-based intervention to promote healthy weight development among young adolescents.

**Methods:**

Participating schools were randomized to Control (n = 25) and Intervention (n = 12). The intervention components to enhance physical activity targeted change through theoretically informed mediators embedded in a social-ecological framework. Accelerometer assessed physical activity (mean count per minute) and self-efficacy, enjoyment, perceived social support from parents, teachers and friends and perceived environmental opportunities were measured by questionnaires at baseline and post-intervention after 20 months among 700 11–13 year-old adolescents (Intervention = 485; Control = 215). The product-of-coefficient test was used to examine mediation.

**Results:**

No mediating effect of any of the hypothesized mediators was identified and gender and weight status did not moderate any mediated effects with the exception of weight status that moderated the mediated effect of enjoyment. Few intervention effects were seen on the mediators, except for a positive change in social support from teachers among girls and the normal weight, and a negative effect on enjoyment and self-efficacy among the overweight. However, change in enjoyment, self-efficacy, perceived social support from friends and environmental opportunities were associated with change in mean count per minute with some variation across the investigated subgroups, and thus show evidence of being potential mediators of physical activity change in adolescents.

**Conclusions:**

While no mediation effects were observed, change in both personal and social-environmental factors predicted change in physical activity behavior. Hence, a social- ecological approach targeting a wide range of determinants to promote change in physical activity holds promise. Overweight and normal weight adolescents may not respond in the same way to school-based physical activity interventions. Therefore, strategies to better reach the overweight seem needed. Future studies should continue to identify mediating and moderation mechanisms in physical activity change in adolescents.

## Background

Regular physical activity (PA) is associated with a decreased risk of health problems in all age groups [1-3]. However, initiation and maintenance of regular PA seem especially important for children in the transition from childhood into adolescence. In this period of life children go through rapid physical and psychosocial changes, and gain more autonomy and decision making power when it comes to health behaviours [4]. At the same time a marked decrease in physical activity level is seen [5,6], and many adolescents in the Western world are not sufficiently active [7]. Therefore, important health benefits can be achieved if PA is encouraged. Recent reviews show that PA in adolescents can be effectively changed through interventions [8,9], but the effects observed are small. This might be due to not targeting potentially effective mechanisms (i.e. theoretical mediating variables) that are substantially related to changes in PA [10]. Mediators are modifiable or intervening variables that specify the causal sequence between an intervention and an outcome (e.g. behaviour) [11]*.* By specifying mediating mechanisms, i.e. what works (i.e. effective intervention components) and what does not work (i.e. ineffective intervention components) in PA interventions, we can prompt future intervention developers to add effective intervention components and remove/adapt ineffective ones.

A recent systematic literature review aimed at examining mediators of overweight prevention interventions in youth, indicated that most publications from PA interventions containing a mediation analyses focused on the mediating effects of personal determinants (e.g. self-efficacy or intention) [12]. Mediating effects of the (perceived) social and physical-environmental factors have not been extensively examined [12]. In addition, studies using an objective measure of PA are called for because it provides a more valid measure of overall PA level in children and young adolescents for whom recall and accuracy of self-report is especially challenging [13].

Intervention effects and their mediators may not be equally effective across subgroups such as gender or groups according to initial weight status [14,15]. One intervention strategy may not cover the diverse needs of various subgroups; i.e. different subgroups may need different types or doses of intervention strategies. Thus, exploring for “whom” working mechanisms of intervention are effective or not is possible with moderation analysis of mediated effect. Moderators are variables that affect the direction and/or strength of the relation between the independent (e.g. the intervention) and the outcome [16]*.* Results from the HEalth In Adolescents (HEIA) study – an intervention designed to promote healthy weight development among young adolescents – show different intervention effects on energy-related behaviours for gender and weight status. Favourably results on sedentary behaviours among girls have been reported [17]. In addition, unpublished findings already show that the intervention did affect overall physical activity expressed as mean count per minute (mcpm) among girls and normal weight adolescents but not among boys and overweight adolescents (unpublished observations, Grydeland M). In addition weight status has been found to moderate effects on determinants of PA [18]. Hence, subgroup intervention and mediating effects by gender and weight status seem important to examine. By conducting a moderation analysis of mediated effect, one examines whether the mediated effect differs across levels of a moderating/grouping variable, and this may help reveal for whom an intervention program is most effective [16].

**Figure 1:**
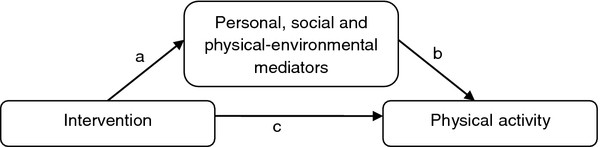
Conceptual mediation model.

The primary aim of the present study was to examine whether changes in personal, social and physical-environmental determinants mediated the effect of change in PA behaviour in the HEIA study (Figure 1). The intervention was developed to change all the underlying constructs, and it was hypothesised that changes in these constructs would act as mediators in predicting changes in PA from baseline (BL) to post-intervention (PI). The secondary aim was to explore whether gender and weight status moderated the mediated effects of the intervention.

## Methods

This study included data on a Norwegian 20 month group randomized controlled trial. The HEIA study aimed at promoting healthy weight development among 11–13 year olds through changes in PA, sedentary behaviours and dietary habits. A detailed description of the design and development has been presented previously [19]. Ethical approval and research clearance was obtained from the Regional Committees for Medical Research Ethics and the Norwegian Social Science Data Service.

### Procedure and participants

**Figure 2:**
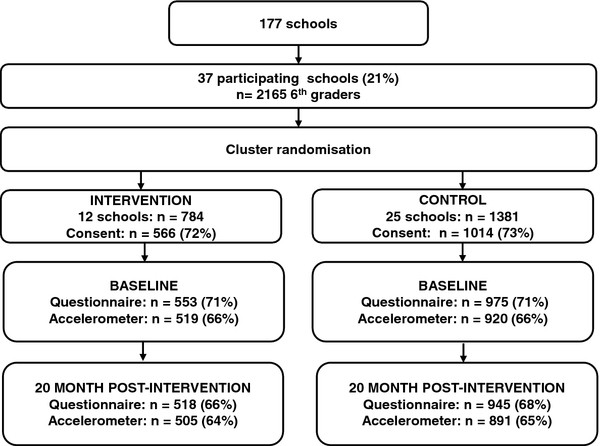
Flow diagram of recruitment, randomization and participation of adolescents in the HEIA study.

Eligible schools had to have more than 40 pupils in 6^th^ grade to participate and be located in the Eastern part of Norway. To meet the criteria the schools were recruited from the largest towns/municipalities in seven counties. Thirty-seven schools were included, and 12 schools were randomly assigned by simple drawing to the intervention group and 25 to the control group (Figure 2). All the 6^th^ graders in these schools (n = 2165) and their parents/legal guardians were invited to participate. Of these, 1580 (73%) returned a signed parental informed consent form. The BL data were collected in 6^th^ grade during fall 2007, the mid-way assessment at the end of 6^th^ grade during spring 2008, and the PI assessment was administered at the end of 7^th^ grade in spring 2009.

At BL, 1528 adolescents completed an Internet-based questionnaire at school, of which 1439 were present and willing to wear an accelerometer and of which 1129 (79%) obtained valid accelerometer data. At PI, 1418 answered the questionnaire, 1396 accelerometers were handed out, and 892 (64%) of those participants provided valid accelerometer data. Included in this paper are 700 participants (485 in Control and 215 in Intervention) that had valid accelerometer data at both BL and PI. As accelerometer data was not collected mid-way, this paper reports on the BL and PI data. In addition, anthropometrics of the adolescents were measured and the adolescents’ self-reported puberty development in a one-page, paper questionnaire (separate versions for boys and girls) at BL and PI [19,20].

### Intervention

The intervention was based on the social-ecological framework and the conceptual model of the HEIA study [19]. It was designed to increase environmental opportunities for PA at school, improve social support, self-efficacy and enjoyment in order to enhance overall level of PA. The PA components described in detail elsewhere [19] included: active commuting campaigns, sports equipment for recess activities, posters in classrooms, one class-room lesson including PA in relation to energy-balance, weekly activity breaks during lessons, 2 inspirational courses for physical education teachers presenting instructional material for PE lessons based on the SPARK Program [21], a computer tailoring program including PA behaviour, fact sheets for parents and yearly kick-off meetings for the teachers. The intervention was implemented by the school staff to increase the feasibility of dissemination of the intervention within the school system in a later phase.

### Measures

#### PA behaviour

The purpose of PA intervention components was to influence overall PA, and therefore mcpm was selected as the outcome variable being a summary measure of total PA. Mcpm was derived from objectively measured PA assessed over 4 consecutive days with the ActiGraph GT1M and CSA model 7164 (ActiGraph, Pensacola, Florida, USA). Since outcomes on mcpm measured by model 7164 and GT1M have shown to differ [22], a free-living validation study of the monitors used in the HEIA study was conducted. As model 7164 was shown to measure 11% higher mcpm than GT1M, a correction factor of 0.9 was applied to the outcomes from model 7164 to be comparable to the GT1M outcome. Data were considered valid if a child had at least three days (including one weekend day) with at least eight hours of activity recorded per day. The procedure for collecting, registering and preparing the data is described elsewhere [23].

#### Mediators

Six theoretically derived personal, social and physical-environmental mediators of the intervention were assessed in the electronic questionnaire based on validated measurements: *Enjoyment of PA*[24] (e.g. “Playing games and sports is the thing I like to do best; Cronbach’s α: at BL/PI; 0.72/0.75) and *self-efficacy related to barriers for PA*[25,26] (e.g. I can be physically active during my free time on most days even if I have the choice to watch TV or play video games instead; α: 0.75/0.78) were both assessed with 5 items. *Perceived social support from parents* (e.g. How often does your mother or father encourage you to play, exercise or do sports? α: 0.70/0.72) was also assessed by 5 items, while *Perceived social support from friends* (e.g. How often do your friends exercise or play sports with you?; α: 0.84/0.84) [27] and *Perceived social support from teachers* (e.g. How often do your teachers encourage you to exercise or play sports?; α: 0.78/0.68) taken from a pilot study within the European Youth Heart Study [28] were assessed by 3 items. *Perceived environmental opportunities* to be physically active at school and during leisure time (e.g. There are other children near my home to go out and play and be physically active with; α: 0.70/0.74) was assessed by 4 items with one added item [27]. All the items for these measures were rated on a 5-point Likert scale coded 1 (“totally disagree”) to 5 (“totally agree”) except for the social support constructs which were phrased “almost never or never”, “one or two times a week”, “three to four times a week”, “almost every day”, “every day”. The computation of the composite scores and results from a separate test-retest study showing acceptable test-retest values (ICC) for these constructs are reported elsewhere [23].

#### Demographic measures, puberty and anthropometrics

Adolescents reported gender and age in the electronic questionnaire. Parental education was reported by the parents on the informed consent form and categorised into 12 years or less, between 13 and 16 years and more than 16 years. Pubertal status was assessed by gender specific versions of the paper questionnaire using the Pubertal Developments Scale (PDS) based on the Pubertal Category Score [29]. The adolescents were categorized into three groups; pre-, early-, or mid/late/post pubertal at baseline [20]. Height and weight were measured by project staff [19,20]. The body mass index cut-off values proposed by the International Obesity Task Force [30] were used to categorize the adolescents as normal weight and overweight/obese.

### Statistical analyses

Independent T-tests and Chi-square tests were conducted to test for differences between the intervention and control group in demographics, mcpm and mediators at baseline, and to test for differences between those lost and those attained at the PI-assessment. If the intervention and control group differed in demographics, mcpm and/or mediator, this specific variable was controlled for in the statistical analysis. Analyses were performed using IBM SPSS Statistics, version 18.0. The alpha level was set at p < .05.

To account for the clustering of the data within schools, Linear Mixed Models analyses with a random intercept for two levels (school (2); individual (1)) were used to analyse the mediated effects. All analyses were adjusted for gender, weight status, parental education level, puberty at BL, and months for assessing accelerometer at BL (September-December) and PI (March-May). A few extreme outliers in the outcome variable (mcpm) were replaced with the mean value + 3SD according to suggested procedures by Field [31]. Assumptions for regression analyses were met. All predicting variables were grand mean centred in order to decrease multicollinearity and to increase interpretation.

To assess mediating effects, the product-of-coefficient test was used [32]. This test consists of (Figure 1): (1) estimating the main effects of the intervention on changes in the outcome variable, wherein the mcpm at PI was regressed on the intervention condition and mcpm at BL (c-coefficient); (2) estimating the effect of the intervention on changes in the potential mediators (a-coefficient) by regressing the PI-values of the mediator onto the intervention condition adjusted for BL-values of the mediator; (3) estimating the independent effect of changes in the potential mediator on changes in mcpm adjusted for the intervention condition (b-coefficient) by regressing the PI-value of mcpm onto the intervention condition, BL-values of mcpm and the PI- and BL-values of the mediator; and (4) computing the product of the two coefficients (a*b), representing the mediated effect. The statistical significance of the mediated effect was estimated by dividing the product-of-coefficient by its standard error. For the calculation of the standard error the Sobel test was used (SE_ab_ = √(a^2^*SE_b_^2^ + b^2^*SE_a_^2^)) [33]. Since mediating effects can still exist without a significant intervention effect on the outcome [34], mediation analyses were also conducted in absence of a significant main effect.

**Figure 3:**
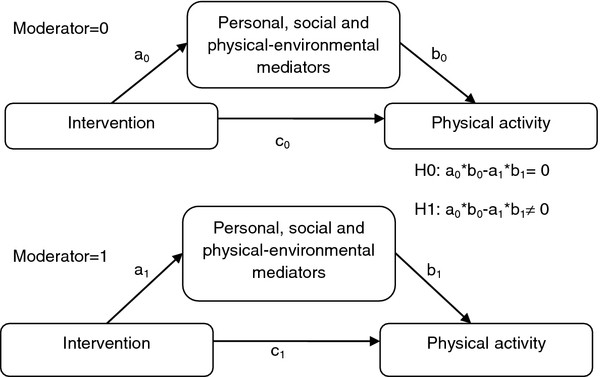
Conceptual model of moderation of a mediated effect.

In addition, the moderating influences of gender and weight status on the mediating effects were studied (Figure 3). For each moderator (e.g. gender), separate mediation models for the subgroups (e.g. boys and girls) were conducted, and product-of-coefficients (ab-coefficient) of both subgroups were compared. If the ab-coefficients in each subgroup are significantly different from one another, there is significant moderation of the mediated effect. To test difference in ab-coefficients between the subgroups for statistical significance, the difference was divided by the pooled standard error (e.g. s_pooled_ = √(s_ab_boys_^2^ + s_ab_girls_^2^)).

## Results

Table 1 shows the baseline characteristics of adolescents in the control and intervention groups. No significant differences in the distribution of the demographic variables between the control and intervention groups were found.

**Table 1:** Age, gender, weight status, parental education and puberty level in control and intervention at baseline

	**Control**	**Intervention**	**p**
**N**	485	215	
**Age** (mean; SD)	11.2 (0.3)	11.2 (0.3)	0.33
**Gender**			
Girls (n;%)	263 (54.2%)	129 (60.0%)	0.20
Boys (n;%)	222 (45.8%)	86 (40.0%)	
**Weight status**			
Normal weight (n;%)	413 (86.0%)	178 (87.3%)	0.72
Overweight/obese (n;%)^a^	67 (14.0%)	26 (12.7%)	
**Parental level of education**			
<= 12 years (n;%)	157 (33.1%)	54 (25.5%)	0.08
13-16 years (n;%)	163 (34.3%)	73 (34.4%)	
> 16 years (n;%)	155 (32.6%)	85 (40.1%)	
**Puberty level**			
Pre pubertal (n;%)	84 (18.7%)	34 (17.1%)	0.84
Early pubertal (n;%)	156 (34.7%)	68 (34.2%)	
Mid/late/post pubertal (n;%)	209 (46.5%)	97 (48.7%)	

^a^ Overweight/obese is presented and treated as one group in the

analyses due to the low proportion of obese (C = 1.9%; I = 2.0%).

More boys than girls (60.9% vs. 47.0%, p < .001) were found among those participating at BL only. Mcpm (mean 526.3 vs. 499.3, p = .007), enjoyment (mean 4.17 vs. 4.06, p = .008), self-efficacy (mean 3.90 vs. 3.82, p = .05) and social support from parents (mean 2.41 vs. 2.32, p = .03) were higher in those with BL data only compared to those with accelerometer data at both time-points. Among those lost to PI, mcpm were higher (mean 541.0 vs. 507.2, p = .04) in the control group compared to the intervention group, but no other differences were found.

The BL/PI wear time (min/day) for the accelerometers was for the control and intervention group 792.5/791.5 and 789.3/771.1, respectively. Table 2 shows the BL- and PI-values and the intervention effect on mcpm (1^st^ step mediation analysis, c-coefficient). There was a borderline significant intervention effect on change in mcpm for all (c = 49.9; 95% CI (-0.4; 100.1), p = .05), and a significant effect among the girls (c = 64.7, 95% CI (5.1; 124.4), p = .03), but not for boys (c = 31.7, 95% CI (35.2; 98.6), p = .35). There was also a significant effect among the normal weight (c = 62.3, 95% CI (9.8; 114.8), p = .02), but a trend for a negative effect among the overweight adolescents (c = -96.1, 95% CI (-211.3; 19.0), p = .12).

**Table 2:** Physical activity (mcpm) at baseline and post-intervention and intervention effect on physical activity

	**Baseline**			**Post-intervention**		**Intervention effect**	
	**Control**	**Intervention**		**Control**	**Intervention**		
	**(n = 485)**	**(n = 215)**		**(n = 485)**	**(n = 215)**		
**Mcpm**	**Mean (SD)**	**Mean (SD)**	**p**	**Mean (SD)**	**Mean (SD)**	**c-coefficient**	**p**
						**(95% CI)**	
All	510.7 (146.0)	473.5 (145.8)	**.002**	563.8 (255.3)	569.8 (251.6)	49.9 (- 0.4; 100.1)	**.05**
Girls	478.3 (127.9)	463.7 (151.3)	.32	506.3 (229.6)	534.9 (234.4)	64.7 (5.1; 124.4)	**.03**
Boys	549.1 (156.8)	488.1 (136.7)	**.002**	631.9 (267.6)	622.2 (268.3)	31.7 (- 35.2; 98.6)	.35
Normal weight	517.2 (142.5)	482.4 (145.9)	**.007**	564.7 (251.7)	584.5 (248.5)	62.3 (9.8; 114.8)	.**02**
Overweight	468.2 (160.0)	406.2 (114.8)	.08	566.3 (282.5)	431.9 (173.0)	- 96.1 (- 211.3; 19.0)	.12

Mcpm = mean count per minute.

Analyses were adjusted for school clustering, gender, pubertal status, months for measuring physical activity, weight category and parental education.

Significant results in bold.

Table 3 shows effect of the intervention on the mediators (2^nd^ step mediation analysis; a-coefficient), the effect of the mediator on mcpm (3^rd^ step mediation analysis; b-coefficient) and the mediated effects of all hypothesized mediators of the intervention effect on mcpm (4^th^ step; a*b). The intervention was effective in changing perceived social support from the teachers (a = 0.12, 95% CI (0.02; 0.21), p = .02) but did not affect the other potential mediators.

**Table 3:** Descriptive potential mediators, intervention effect on mediators, effect of mediators on mcpm and mediated effect

	**Baseline**			**Post-intervention**		**Mediation analyses**					
	**Control**	**Intervention**		**Control**	**Intervention**						
	**(n = 475-480)**	**(n = 210-215)**		**(n = 483-485)**	**(n = 210-215)**						
**Mediators**	**Mean (SD)**	**Mean (SD)**	**p**	**Mean (SD)**	**Mean (SD)**	**Intervention effect on mediator**	**p**	**Effect mediator**	**p**	**Mediated effect**	**p**
								**on mcpm**		**ab (95% CI)**	
						**a (95% CI)**		**b (95% CI)**			
Enjoyment	4.09 (0.78)	4.01 (0.76)	.21	3.97 (0.82)	3.86 (0.81)	- 0.07 (-.20; 0.06)	.30	**32.78 (10.03; 55.53)**	**.005**	- 2.31 (-6.86; 2.25)	.32
Self-efficacy	3.81 (0.77)	3.83 (0.75)	.70	3.84 (0.83)	3.73 (0.79)	- 0.15 (-.31; 0.01)	.06	**42.56 (19.07; 66.06)**	**<.000**	- 6.42 (-13.83; 0.98)	.09
Social support friends	2.92 (1.00)	2.86 (1.07)	.47	2.73 (1.00)	2.70 (0.86)	- 0.02 (-0.16; 0.13)		**41.56 (21.58; 61.53)**	**<.000**	- 0.67 (-6.74; 5.40)	.83
Social support parents	2.30 (0.78)	2.37 (0.76)	.34	2.23 (0.80)	2.19 (0.74)	- 0.03 (-16; 0.10)	.62	9.60 (-17.30; 36.50)	.49	- 0.30 (-1.69; 1.11)	.68
Social support teachers	1.67 (0.72)	1.79 (0.83)	.08	1.43 (0.57)	1.56 (0.66)	**0.12 (0.02; 0.21)**	**.02**	- .13 (-31.81; 31.57)	.99	- 0.02 (-3.75; 3.73)	.99
Environmental opportunities	4.22 (0.80)	4.26 (0.82)	.54	4.08 (0.89)	4.18 (0.89)	0.11 (-0.04; 0.26)	.15	15.53 (-5.61; 36.67)	.15	1.72 (153; 4.98)	.29

Mcpm = mean count per minute.

Baseline differences between the potential mediators were tested with independent t-test.

Significant results in bold.

Changes in enjoyment, self-efficacy and social support from friends were significantly and positively associated with change in mcpm. No relationships were found between changes in social support from parents and teachers, or environmental PA opportunities and mcpm. None of the hypothesized mediators mediated the intervention effect on mcpm.

Table 4 shows the separate mediation models by gender. Among girls, but not boys, the intervention was effective in changing social support in teachers (a = 0.18; 95% CI (0.05; 0.31), p = .01). In addition the b-coefficient analyses showed significant positive associations between changes in self-efficacy and environmental opportunities and changes in mcpm among girls. Change in enjoyment and social support from friends was associated with enhanced PA behaviour among boys. No significant moderation of gender on the mediated intervention effects was found, indicating that the working mechanisms of the interventions did not differ between boys and girls.

**Table 4:** Effect on mediators, effect of mediators on mcpm, mediated effect and moderated mediation of gender

**Mediators**	**Gender**	**Intervention effect**	**p**	**Intervention effect**	**p**	**Mediated effect**	**p**	**Moderated mediation**	**p**
		**on mediator**		**on mcpm**		**ab (95% CI)**			
		**a (95% CI)**		**b (95% CI)**				**Δab (95% CI)**	
Enjoyment	Girls	- 0.12 (-0.32; 0.07)	.19	24.40 (-4.73; 53.54)	.10	- 3.03 (-8.62; 2.56)	.29	- 3.81 (-10.58; 2.96)	.27
	Boys	0.02 (-0.20; 0.24)	.85	**37.06 (1.18; 72.93)**	**.04**	0.78 (-7.24; 8.80)	.85		
Self-efficacy	Girls	- 0.13 (-0.32; 0.06)	.18	**64.37 (33.84; 94.89)**	**<.000**	- 8.17 (-20.38; 4.04)	.19	*-* 5.06 (-15.28; 5.16)	.33
	Boys	- 0.14 (-0.36; 0.07)	.19	21.76 (-14.13; 57.64)	.23	- 3.11 (-10.00; 3.80)	.38		
Social support friends	Girls	- 0.02 (-0.16; 0.21)	.81	20.51 (-5.14; 46.15)	.12	0.46 (-3.33; 4.25)	.81	2.47 (-7.93; 12.86)	.64
	Boys	- 0.03(-0.28; 0.21)	.80	**61.71 (30.24; 93.17)**	**<.000**	- 2.01 (-17.09; 13.08)	.79		
Social support parents	Girls	0.01 (-0.16; 0.17)	.91	- 2.99 (-38.40; 32.41)	.88	- 0.03 (-0.59; 0.54)	.99	1.10 (-4.77; 6.97)	.71
	Boys	- 0.03 (-0.26; 0.21)	.80	27.21 (-14.14; 68.56)	.20	- 1.13 (-9.96; 7.70)	.80		
Social support teachers	Girls	**0.18 (0.05; 0.31)**	**.01**	- 8.48 (-48.86; 31.91)	.69	- 1.53 (8.90; 5.83)	.68	- 2.10 (-8.03; 3.83)	.49
	Boys	0.06 (-0.11; 0.22)	.50	9.90 (-40.89; 60.69)	.70	0.56 (-2.72; 3.85)	.74		
Environmental opportunities	Girls	0.10 (-0.08; 0.28)	.25	**31.41 (3.76; 59.06)**	**.03**	3.23 (-2.91; 9.37)	.74	3.84 (-1.96; 9.65)	.19
	Boys	0.17 (-0.10; 0.43)	.21	-3.72 (-35.63; 28.19)	.82	- 0.62 (-5.97; 4.73)	.82		

Mcpm = mean count per minute.

Significant results in bold.

Table 5 shows the separate mediation models by weight status. Among normal weight, but not overweight adolescents, the intervention was effective in changing social support in teachers (a = 0.13, 95% CI (0.03; 0.23), p = 0.01). A negative intervention effect on enjoyment (a = -0.47, 95% CI (-0.90; -0.04), p = .03) and self-efficacy (a = -0.63, 95% CI (-1.03; -0.23), p = 0.002) was seen among overweight adolescents. Significant positive associations between changes in self-efficacy, social support from friends and environmental opportunities and changes in mcpm among the normal weight were found. Among the overweight, the only significant association seen was between changes in enjoyment and mcpm. Weight status did moderate the mediating effect of enjoyment, indicating that the mediating effect of enjoyment on the intervention differed among normal weight and overweight adolescents. No other mediating effects were identified in the normal weight or overweight adolescents.

**Table 5:** Effect on mediators, effect of mediators on mcpm, mediated effect and moderated mediation of weight-status

**Mediators**	**Weight status**	**Intervention**	**p**	**Effect mediator on mcpm**	**p**	**Mediated effect**	**p**	**Moderated mediation**	**p**
		**effect on mediator**				**ab (95% CI)**			
		**a (95% CI)**		**(b; 95% CI)**				**Δab (95% CI)**	
Enjoyment	Normal weight	- 0.01 (-0.17; 0.16)	.94	24.40 (-0.15; 48.94)	.05	- 0.14 (-3.90; 3.61)	.94	**45.99 (27.44; 64.54)**	**<.000**
	Overweight	**- 0.47 (**-**0.90; -0.04)**	**.03**	**97.65 (35.34; 159.95)**	**.003**	-46.13 (-95.73; 3.46)	.07		
Self-efficacy	Normal weight	- 0.09 (-0.26; 0.08)	.30	**46.73 (21.25; 72.22)**	**<.000**	-4.05 (-11.91; 3.82)	.31	- 0.30 (-15.97; 15.37)	.97
	Overweight	**- 0.63 (**-**1.03; -0.23)**	**.002**	5.97 (-54.87; 66.80)	.85	- 3.75 (-41.48; 33.98)	.85		
Social support friends	Normal weight	-0.01 (-0.16; 0.14)	.90	**45.23 (23.47; 66.98)**	**<.000**	- 0.42 (-7.34; 6.50)	.91	3.72 (-4.84; 12.27)	.40
	Overweight	- 0.15 (-0.67; 0.36)	.54	26.68 (-24.53; 77.88)	.30	- 4.13 (-19.47; 11.21)	.60		
Social support parents	Normal weight	- 0.03 (-0.06; 0.10)	.70	7.00 (-22.11; 36.10)	.64	- 0.18 (-1.34; 0.97)	.76	0.41 (-2.71; 3.53)	.80
	Overweight	- 0.03 (-0.36; 0.31)	.88	23.61 (-51.40; 98.63)	.53	- 0.59 (-8.57; 7.38)	.88		
Social support teachers	Normal weight	**0.13 (0.03; 0.23)**	**.01**	- 9.21 (-43.69; 25.27)	.60	- 1.20 (-5.78; 3.38)	.61	- 6.48 (-13.88; 0.92)	.09
	Overweight	0.12 (-0.19; 0.43)	.43	43.32 (-4.41; 128.05)	.31	5.28 (-11.20; 21.76)	.53		
Environmental opportunities	Normal weight	0.11 (-0.06; 0.29)	.19	**24.75 (1.62; 47.87)**	**.04**	2.84 (-2.07; 7.74)	.26	5.40 (-1.03; 11.82)	.10
	Overweight	0.10 (-0.35; 0.56)	.65	-24.56 (-79.42; 30.30)	.38	2.56 (-14.87; 9.75)	.68		

Mcpm = mean count per minute.

Significant result in bold.

## Discussion

None of the personal, social or physical-environmental constructs targeted in the intervention were found to mediate the PA outcome (mcpm). Regarding enjoyment and self-efficacy as mediators, our findings partly contrast previous results. One the one hand, a mediation effect of enjoyment and strong evidence for a mediation effect of self-efficacy have been observed [12,35-38]. On the other hand, results from other studies support our findings for these constructs [12]. Regarding perceived social support and physical-environmental opportunities as mediators, our findings are in line with previous research which has revealed no clear evidence for mediating effects for social support or environmental measures [12]. The lack of mediation findings in this study was mainly due to the lack of intervention effects on the potential mediators. In addition, gender did not moderate any of the mediation effects of the intervention effect on mcpm, but a moderated mediation effect of weight status on enjoyment was observed.

The only mediator positively affected by the intervention was perceived social support from teachers. The subgroup analyses revealed that this effect was present in girls and normal weight adolescents only*.* However, since changes in teacher support were not associated with change in mcpm (non-significant b-coefficient) teacher support did not stand out as a mediator of the intervention effect. Teacher support is less studied than support from friends and parents [12], but teachers are in a position to reach most adolescents and thus may play the role of possible change facilitators in school-based interventions. Haerens et al. [38] found a positive association between teachers’ social support and school related sports activities*.* One explanation for the lack of significant association between teacher support and PA in our study could be that the objectively assessed overall PA, which covers both within school and out-of school activity, has deflated the effect of teacher support.

Among overweight adolescents, we found a negative intervention effect on enjoyment and self-efficacy. This is in line with previous mid-way assessment results and indicates that the intervention activities did not meet the needs of those who are overweight [18]. Despite our focus on changing behaviours and not weight status, the children were aware of the main purpose of the intervention (to promote a healthy weight development). Hence, overweight adolescents may have felt uncomfortable during the intervention, which eventually could have led to a psychological reactance reducing their enjoyment and self-efficacy for PA [39]. Alternatively, social comparison processes between normal weight and overweight adolescents might have led to unfavorable self-perceptions and stigmatization [40,41]. However, the negative impact on self-efficacy may also reflect a more realistic interpretation of barriers to PA among the overweight prompted by the intervention.

The non-significant intervention effect on the other mediators (social support from friends and parents, perceived environmental opportunities) and PA can have several explanations. First, ineffective intervention strategies and/or insufficient implementation of those strategies may explain the limited effect on the potential mediators in general. Previous process evaluation in a larger sample showed that only 31% of the adolescents reported being exposed to/participated to a high degree in the PA intervention components in the last school year [18]. The same result was observed in the current sample (data not shown). Both the length of the intervention and the responsibility given to teachers in implementing the intervention could have led to great variation in implementation quality between the schools, and could probably partly explain the limited effects on both the mediators and the outcome.

Second, a possible mismatch could have existed between the specific intervention strategies and potential mediators they were meant to target. Generally, the intervention components were developed to facilitate active play at schools, active transport to and from schools and more daily-living PA. Several of these PA behaviours might represent more or less habitual forms of activity and are thus likely to be automatized and facilitated by environmental and situational cues [42] rather than by changes in cognitions such as self-efficacy and social support. Self-efficacy and social support might be constructs that are better able to predict conscious- and intention driven activities such as typical sport activities.

Third, the limited effect on the mediator could also be explained by relatively high BL values in several of the constructs (Table 3) and/or an insensitivity of the measurement instruments to detect change. The mediator measures were directed towards PA in general. By phrasing the items towards the specific contexts targeted, they might have been more sensitive to change. This, however, would have extended the length of our questionnaire, thus causing a threat to the overall validity. It could also well be that an intervention by measurement effect may have occurred [34], meaning that the intervention affected the response to the items making it less likely to detect an effect.

Despite a limited effect on the potential mediators, changes in personal, social and physical environmental determinants were associated with changes in PA with differences among gender and weight status groups. Self-efficacy (girls and normal weight only), perceived environmental opportunities (girls only), social support from friends (boys and normal weight only) and enjoyment (boys and overweight only) were associated with PA change. Hence, these factors hold potential to be included in future interventions. The findings add support to previous studies in which a change in enjoyment and self-efficacy has been found to be positively related to self-reported PA in intervention studies [35-37,43]. Social support from friends has been shown to mediate accelerometer assessed moderate-to-vigorous PA in girls [44], and our results indicate that it might be a relevant mediator for boys as well. The result for perceived environmental opportunities shows that the environment does play a role, and the mediating role of environmental factors on adolescents’ PA seems important to examine further.

### Strengths and limitations

This study has both strengths and limitations. In addition to the randomized controlled design, the intervention was theoretically informed and included mediators representing both the social and physical environmental domains as well as the personal domain. All the mediators showed acceptable internal consistency. Moderated mechanisms were explored and PA was assessed objectively in a sample of adolescents that was younger than previously examined [12].

However, accelerometers assessed PA are not able to capture water activities, record cycling, upper body movements, carrying a load correctly or detect context specific changes in PA, and using logs in addition to accelerometers could have compensated for these drawbacks [45,46]. The larger than expected drop-out [19], could have caused a loss of power and may have influenced the results, especially among the overweight. There exists several sets of BMI reference data that are intended to define childhood overweight [47]. Therefore, we cannot rule out the possibility that the results for the moderated mediation of weight status might have been slightly different applying for example the cut off values for the World Health Organizations’ growth curves or the Center for Disease Control. Still, within the HEIA study we choose to use the IOTF’s criteria for defining overweight/obesity to allow for comparing prevalence data across nations [47,48].

In addition, those lost to the PI assessment were more likely to be boys, but in the analyses this was compensated for by adjusting for gender. Among those lost to PI, higher values for mcpm were found in the control group compared to the intervention group, but this was the case in the study sample also (Table 2) and adjusted for in the analyses.

## Conclusions

In conclusion, no mediated effects on PA change through the mediators could be observed. This was mainly due to the lack of intervention effect upon most of the hypothesized mediators, even though the HEIA intervention did have a borderline significant effect on mcpm in the total sample, and a significant effect was seen among girls and normal weight adolescents (unpublished observations, Grydeland M). Both personal, social and physical-environmental factors were identified as potential mediators of objectively measured PA, and the results indicate that enjoyment and self-efficacy, social support from friends and perceived environmental opportunities seem worthwhile to target in future interventions. However, unfavourable results of the intervention were revealed for the overweight group.

Further mediation analyses of PA change among adolescents based on a social-ecological framework including a broad based set of mediators seem warranted. Strategies better able to affect mediators and efforts to secure sufficient strength of the implementation should be emphasized. This would make it more likely to obtain changes in the mediators and, consequently, to detect a relationship between change in the mediators and change in PA. When developing prevention studies targeting energy-related behaviours it seems necessary to include strategies tailored to gender and weight status.

## Abbreviations

PA: Physical activity; Mcpm: Mean count per minute; BL: Baseline; PI: Post-intervention.

## Competing interests

The authors declare that they have no competing interests.

## Authors’ contribution

IHB conducted the statistical analyses assisted by MM van S, wrote the first draft of the manuscript and made the greatest contribution to the paper. MG, MB, NL, LFA, SA and YO participated in designing the study, project planning and data collection. All authors have critically revised the manuscript, and read and approved the final version of the manuscript.

## Pre-publication history

The pre-publication history for this paper can be accessed here:

http://www.biomedcentral.com/1471-2458/12/814/prepub
